# Evolution Model of Spatial Interaction Network in Online Social Networking Services

**DOI:** 10.3390/e21040434

**Published:** 2019-04-24

**Authors:** Jian Dong, Bin Chen, Pengfei Zhang, Chuan Ai, Fang Zhang, Danhuai Guo, Xiaogang Qiu

**Affiliations:** 1 College of System Engineering, National University of Defense Technology, Changsha 410073, China; 2 Computer Network Information Center, Chinese Academy of Sciences, 4th South Fourth Road Zhongguancun, Beijing 100190, China; 3 University of Chinese Academy of Sciences, 19th Yuquan Road, Beijing 100049, China

**Keywords:** city interaction network, evolution model, preferential attachment, WeChat, maximum likelihood

## Abstract

The development of online social networking services provides a rich source of data of social networks including geospatial information. More and more research has shown that geographical space is an important factor in the interactions of users in social networks. In this paper, we construct the spatial interaction network from the city level, which is called the city interaction network, and study the evolution mechanism of the city interaction network formed in the process of information dissemination in social networks. A network evolution model for interactions among cities is established. The evolution model consists of two core processes: the edge arrival and the preferential attachment of the edge. The edge arrival model arranges the arrival time of each edge; the model of preferential attachment of the edge determines the source node and the target node of each arriving edge. Six preferential attachment models (Random-Random, Random-Degree, Degree-Random, Geographical distance, Degree-Degree, Degree-Degree-Geographical distance) are built, and the maximum likelihood approach is used to do the comparison. We find that the degree of the node and the geographic distance of the edge are the key factors affecting the evolution of the city interaction network. Finally, the evolution experiments using the optimal model DDG are conducted, and the experiment results are compared with the real city interaction network extracted from the information dissemination data of the WeChat web page. The results indicate that the model can not only capture the attributes of the real city interaction network, but also reflect the actual characteristics of the interactions among cities.

## 1. Introduction

With the rapid development of the Internet, smart phones, and information technology, online social networking services such as Facebook, Twitter, Sina Weibo, and WeChat have developed rapidly. These platforms facilitate the interactions among users and accelerate the dissemination of emotions and opinions contained in the information. Meanwhile, these platforms provide a rich source of social media including geospatial information for the research of social networks [1,2,3,4]. The interactions of users in social networks usually manifest as the viewing and forwarding of information. More and more research shows that geographical space, which seems to be a bridge between online and offline, affects the interactions of users in social networks [5,6,7].

Spatial interaction is the process whereby entities at different points in physical space make contacts, demand/supply decisions, or locational choices [8]; for example, trade in goods among different countries or regions, human migration among cities or countries, and people in different cities communicating with each other by phone or social media software. In social networks, spatial interactions are formed by users who belong to different spatial locations through viewing and forwarding information. Naturally, spatial interactions can be described by complex network [9], where nodes represent spatial locations, which can be cities, provinces, or countries, and edges represent interactions of entities in different spatial locations. The research on the characteristics of the spatial interaction network in social networks and their evolutionary mechanisms is of great significance for providing location-based business services, planning and managing communication network facilities, and formulating regional economic development policies. In addition, the results also can be used to improve the performances of several types of applications in various fields, such as social network analysis [10] and affective computing [11,12,13].

The existing network evolution models mainly include the random graph models (RGM) [14,15,16], generated network models (GNM) [17,18], and data-driven network models (DDNM) [19,20,21]. Random graph models, such as Poisson random graphs and generalized random graphs, attempt to apply the connecting probability and changing strategy of the edge to a certain number of nodes to generate a random network that meets specific statistical characteristics (such as average degree, degree distribution, joint degree distribution, and degree-degree correlation). Generated network models, such as preferential attachment models and their variants, try to generate a network that reflects certain characteristics of the real network (such as a power-law distribution, small-world characteristics, and homogeneity) through certain node-adding, edge-adding, and edge-changing rules from simple graphs (regular graphs). These two widely-used models can usually generate networks with some characteristics of the real network, but they cannot satisfy multiple characteristics at the same time. Moreover, these models usually do not consider the geospatial characteristics of networks, making it difficult to describe the evolution process of spatial interaction network.

Generally, distance and location are the two important factors of geospatial characteristics. On the one hand, it is found that the interaction frequency among users has a distance decay effect. People tend to communicate more with friends who are close to them geographically, while users who are far away from each other are less likely to interact [22,23,24,25]. On the other hand, the behaviors of people living in similar geographical locations, such as the same city, often show similarities, while people in different geographical locations will have different behavior patterns due to economic and cultural differences, thus affecting the information interactions among regions [22].

Gravity laws are commonly found in spatial interaction networks such as crowd flow networks, population migration networks, and commodity trade networks. Thus, a gravity model for spatial interaction is proposed by analogy with the law of universal gravitation. The gravity model provides an estimate of the traffic between two or more regions (such as the number of trips and the quantity of commodity trade). In a spatial interaction network, the gravity model can be interpreted as the frequency of interactions between two nodes. The frequency is proportional to the strength of the two nodes and inversely proportional to the power of the distance between the two nodes. The gravity model has become a classic model for interpreting and predicting the interactions of spatial networks and is widely used in many fields including transportation planning [26], population migration [27,28], international trade [29,30], and disease transmission [31]. Although the gravity model is simple, intuitive, easy to calculate, and involves geographical factors, it lacks a rigorous theoretical foundation. In addition, the gravity model is deterministic and cannot explain the fluctuation of the interaction between two nodes in the spatial interaction network [32]. Therefore, this kind of static estimation is not suitable for describing the evolution of spatial networks.

This paper proposes a spatial interaction network at the city level, which is called the city interaction network. We study the evolution mechanism of the city interaction network formed in the process of information dissemination in social networks, where nodes represent cities and edges represent interactions among cities. We consider the evolution model of the city interaction network from the perspective of the edge, that is how each edge is added to the city interaction network. A evolution model for describing the interactions among cities is established. The evolution model consists of two core processes: the edge arrival and the preferential attachment of the edge. The edge arrival model arranges the arrival time of each edge; the model of preferential attachment of the edge determines the source node and the target node of each arriving edge. Six preferential attachment models (Random-Random, Random-Degree, Degree-Random, Geographical distance, Degree-Degree, Degree-Degree-Geographical distance) are built, and the maximum likelihood approach is used to do the comparison. Finally, the evolution experiments using the optimal model (Degree-Degree-Geographical distance) are conducted, and the experiment results are compared with the real city interaction network extracted from the information dissemination data of the WeChat web page.

Preferential attachment of edges: The preferential attachment model assumes that when a new node joins the network, it creates a constant number of edges, where the selection of the target node for each edge is proportional to the degree of the node [33]. In addition to degree, the node age and geographic distance of the edge can be applied to the preferential attachment model [34]. This paper considers the evolution of the network from the perspective of the edge. Therefore, when an edge is added to the network, the source node and the target node are selected according to preferential attachment of edges.

Evaluation by the maximum likelihood: The maximum likelihood approach is usually used to compare a series of models numerically and select the best model (and parameters) to interpret the data [35]. As our understanding of real-world networks improves, likelihood remains unchanged, while the generative models improve to incorporate the new understanding. Success in modeling can therefore be effectively tracked [34]. The maximum likelihood approach is widely used to estimate network model parameters [35,36,37] and select the optional model [34,38]. Therefore, this paper uses the maximum likelihood approach to evaluate and compare different network evolution models based on empirical data.

WeChat: WeChat is one of the most popular social networking platforms in China. As of the second quarter of 2016, WeChat has covered more than 94% smart phones in China, with 0.8 billion monthly active users. WeChat has powerful social functions and a large number of users, and WeChat has integrated almost all aspects of people’s lives, including payment, location-based services, shopping, games, and entertainment. Therefore, WeChat is an appropriate system to study the characteristics and evolution mechanism of the spatial interaction network in social networks.

The rest of this paper is organized as follows: the second section introduces the dissemination data of the WeChat web page and constructs the city interaction network. The third section introduces the evolution model of the city interaction network. In the fourth section, the maximum likelihood method is used to evaluate the six preferential attachment models and to select the optimal model and parameters. In the fifth section, the optimal model is used for network evolution, and the obtained evolutionary network is compared with the real city interaction network. The potential biases and model extension are discussed in the sixth section, and the seventh section is the conclusion.

## 2. Preliminaries

### 2.1. Dataset

WeChat provides three basic functions: instant messaging (including single and group chat), moments (where users publish, comment, and forward information), and official accounts (including subscription accounts and service accounts). Users can interact with their friends by posting text, voice, pictures, emoticons, location, video, web links, and other information. This paper studies the dissemination data of the WeChat web page (HTML5) collected by third-party service companies. The recording process of the WeChat web page data can be described as: when a web page with a certain theme is created and published by the creator through the official accounts, the content of this web page can be viewed by other users. Users who view the web page can send it to their moments or WeChat friends, or not forward it. Thus, the users who view (or forward) and the users who are viewed (or forwarded) are recorded.

The dissemination data of WeChat web page were obtained, and the time span of the data was from 2–8 July 2016. There were 622,637 records in total, and each record can be represented by a six-tuple <pageID, sourceID, targetID, type, time, ip>, where pageID represents the unique identity of the web page, sourceID and targetID represent the unique identity of the user, type represents the behavior type of target, including viewing and forwarding, time represents the time when the behavior of targetID occurs, and ip represents the IP address of targetID. In order to protect the privacy of users, web page identity and user identity were anonymized.

### 2.2. City Interaction Network

Most of the researches related to geography use self-reported data to identify the location of users, which is often inaccurate. By locating users with IP addresses, the errors of self-reported data can be avoided. Song et al. analyzed several large IP address databases, including the Chunzhen IP address database, the Taobao IP address database, the Sina IP address database, and the Baidu IP address database [39]. They found that the four IP address databases were quite different, and when the administrative division level was lower, the coverage rate and coincidence rate of IP address databases would decrease, while the data availability would also decrease. However, considering the coverage rate and coincidence rate of the four IP address databases, they believed that the credibility of the Taobao IP database was the highest. Therefore, the Taobao IP address database was used in our work to locate the IP address in the data to the corresponding cities in China. Finally, the IP address in the data was located in 34 provincial divisions of China (including 23 provinces, 4 municipalities, 5 autonomous regions, and 2 special administrative regions), a total of 372 cities. The number of cities corresponding to each provincial division is shown in Table 1.

**Figure 1:**
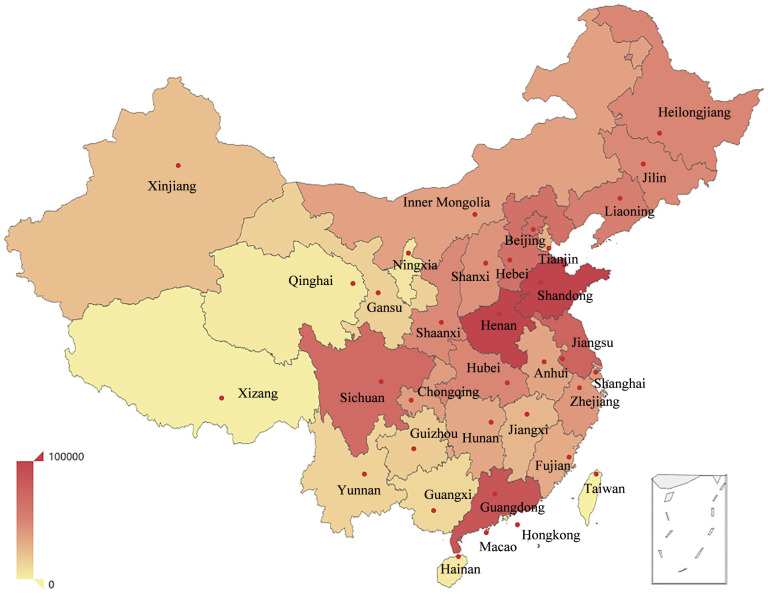
The active frequency of users in 34 provincial divisions of China. The transition of colors from red to yellow indicates the reduction of active frequency, and the corresponding data of each color are given by the color bar in the lower left corner.

Figure 1 shows the active frequency of users in each provincial division. The active frequency of a province is the number of users located in that province. The active frequency was more in the east and less in the west. The top three provincial divisions with the highest frequency were Shandong, Henan, and Guangdong, and the active frequency of Xizang, Xinjiang, and Taiwan was low. This fully reflects that information interaction is affected by political, economic, cultural, geographical, and demographic factors.

**Figure 2:**
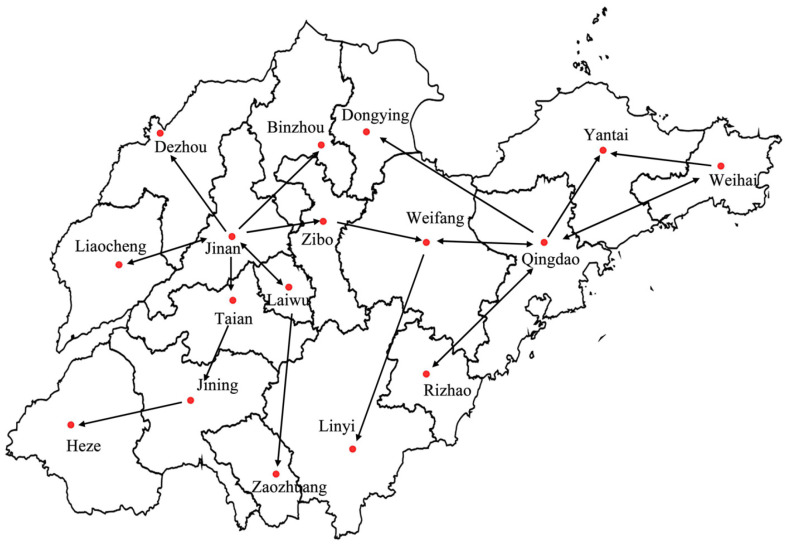
Schematic diagram of the city interaction network in Shandong province. Red dots represent the nodes of the network, and black arrows represent the directed edges of the network. The arrows start from the source node and point to the target node. The bidirectional arrow indicates that the two nodes are source and target nodes of each other.

Based on the data of the web page dissemination in WeChat, the city interaction network $G_{t}=\left(V,E_{t},W_{t}\right)$ can be constructed. $G_{t}$ is a dynamic directed network, $V=\{v_{1},v_{2},v_{3},\cdots ,v_{N}\}$ is the set of nodes in the network, representing cities of China, and the number of nodes is *N*; $E_{t}=\left\{e_{1},e_{2},e_{3},\cdots ,e_{M_{t}}\right\}$ is the set of edges of the network from Time 0–*t*, representing the interactions among cities, and the number of edges is $M_{t}$; $W_{t}=\left\{w_{1},w_{2},w_{3},\cdots ,w_{M_{t}}\right\}$ is the weight set of edges in the network from Time 0–*t*, representing the number of interactions among cities. The dynamics of the city interaction network $G_{t}$ is reflected in the changes of the edge and weight. We took the cities in Shandong province as an example to elaborate the construction process of the city interaction network. At t=0, $G_{t}$ is a network containing only 17 isolated nodes (the number of cities in Shandong province). When a WeChat web page is published by a user in Jinan and users in Dezhou view or forward this web page, then a directed edge from Jinan to Dezhou is established. The weight of the directed edge is the number of Dezhou users viewing the web page. With the dissemination of the web page, it was assumed that the interaction network one day later is as shown in Figure 2. At this time, the number of nodes in the interaction network was N=17, and the number of edges was $M_{t}=22$ (bidirectional edges are denoted as two edges), where t=1 (day). The city interaction network in this paper allows self-connected edges, which represents the interactions in the same city.

Take the starting time of data (2 July 2016 00:00) as the time t=0, and construct the city interaction network. The time span of the network is *T*. Table 2 lists the basic properties of the network $G_{T}$, including the number of nodes, number of edges, number of self-connected edges, average degree of nodes, density, average clustering coefficient, and average shortest path length.

According to the basic properties of the network $G_{T}$ listed in Table 2, an overall understanding of the interaction among cities was obtained through the dissemination of WeChat web page. The network involved 372 nodes and 30,438 edges, which indicates that not every two nodes had connected edges. On average, each node only had connections with 163.65 nodes, and the density of the network was only 0.22. It can be seen that although WeChat has a large number of users in China and covers all cities, each city will not interact with all other cities in the short term. The average shortest path length of the network was 1.73, which means that the average hop from one node to another node was 1.73. There were 353 self-connected edges in the network, and only 19 nodes had no self-connected edges. A total of 622,637 interaction records were recorded, among which, 350,578 records were the interactions in the same city, accounting for 56%. It can be seen that users were more inclined to interact with users in the same city.

**Figure 3:**
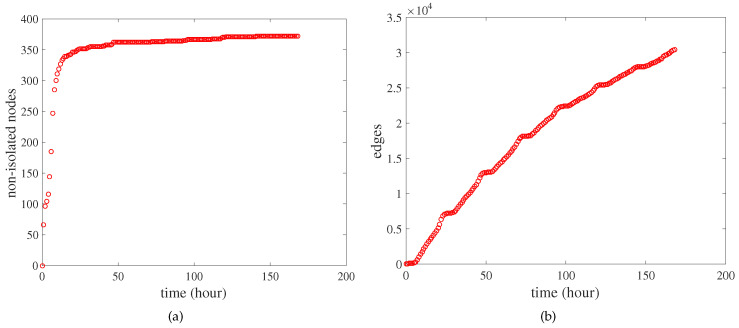
The number of non-isolated nodes and the number of edges in the city interaction network as a function of time. (**a**) The number of non-isolated nodes in the city interaction network as a function of time. (**b**) The number of edges in the city interaction network as a function of time. Each data point in the figure represents the number of non-isolated nodes (or edges) in the city interaction network from t=0 to the current time. The time interval between two data points is one hour.

Figure 3 shows the number of non-isolated nodes and the number of edges in the city interaction network as a function of time. Figure 3a shows the number of non-isolated nodes in the city interaction network as a function of time. Non-isolated nodes represent the nodes that have interacted with other nodes. In the initial stage, the number of non-isolated nodes grew rapidly, and the growth became slow until the number of nodes was close to *N*. Figure 3b shows the number of edges in the city interaction network as a function of time. The number of edges in the network kept increasing, but due to the limitation of the number of nodes, the growth of the number of edges gradually slowed down. In the case where the number of non-isolated nodes in the network was almost constant, the number of edges still kept growing. This also reflects the limitations of the evolution of the city interaction network from the perspective of nodes.

### 2.3. Notation

Let *Z* denote the set of edges to be added to the network, t(z),z∈Z the time when an edge *z* is added to the network, and $z_{u,v}^{t}$ an edge *z* added to the network at time *t*, and its source node and target node are connected to node *u* and node *v* respectively. Let $k_{t}\left(v\right)$ denote the degree of node *v* at time *t* and d(u,v) denote the geography distance between node *u* and node *v*.

## 3. Evolution Model

We consider the evolution model of the city interaction network from the perspective of the edge. The model consists of two core processes: the edge arrival and the preferential attachment of the edge. The edge arrival determines the arrival time of each edge; the preferential attachment of the edge determines the source node and the target node of each arriving edge.

For an edge *z*, it is composed of a node pair:

(1) z=(u,v),u,v∈V,

where *V* represents the node set and does not change with the network evolution. Assuming that the arrival time of the edges is a function of time in Δt, then the arrival time of each edge in Δt will be arranged, and all edges can be expressed in the time sequence according to the arrival time:

(2) $$Z=z^{t_{1}},z^{t_{2}},\cdots ,z^{t_{C}},$$

(3) $$t_{1}⩽t_{2}⩽\cdots ⩽t_{C},$$

where *C* is the length of the sequence *Z*, and Formula (3) guarantees the time-ordered arrival of the edges.

Select the source node *u* and the target node *v* from node set *V* according to a certain preferential attachment for the edge arriving at time *t*:

(4) $$P\left(z_{u,v}^{t}\right)∼X\left(\Theta \right),$$

where X(Θ) represents a distribution function and Θ is the parameter of the distribution function. Finally, the network evolution is realized by updating the edge and weight. The edge arrival and preferential attachment of the edge are described in detail below.

### 3.1. Edge Arrival

**Figure 4:**
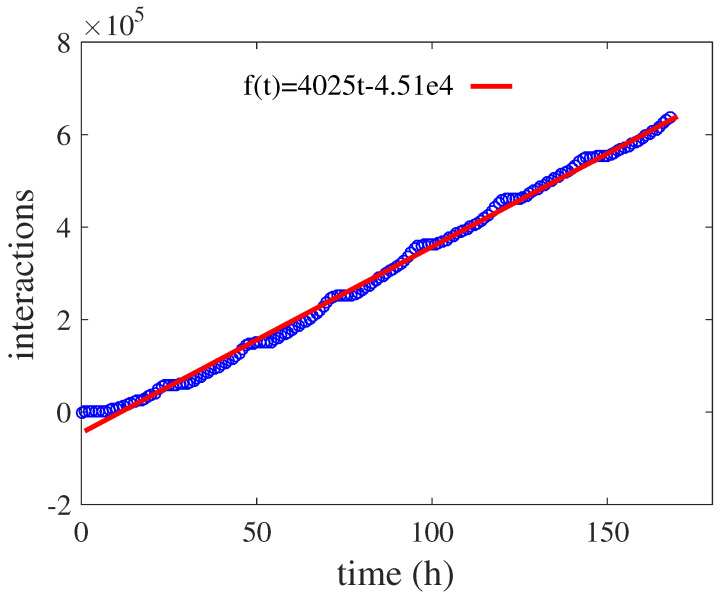
The interaction quantity among cities of the data as a function of time. Each data point represents the interaction quantity among cities from time t=0 to the current time, and the red line is the fitting for the function; the fitting expression is given in the figure.

Figure 4 shows the interaction quantity among cities of the data (each record represents an interaction) as a function of time. In the figure, each data point represents the interaction quantity among cities from time t=0 to the current time, and the red line is the fitting of the function. It can be seen from the figure that the interaction quantity was a linear function of time, which satisfies f(t)=4025t−4.51e4, and the time unit is hours. Since each edge represents the interaction among nodes, f(t) can be used to describe the number of arriving edges. Thus, the number of edges added to the network per unit time is a constant ε=4025, and the time interval for each arriving edge is $t_{i}-t_{i-1}=1/\varepsilon ,i=2,3,\cdots ,C$. Let the time of the first arrived edge be $t_{1}=0$, so that the time of each arriving edge is determined.

### 3.2. Preferential Attachment of the Edge

In this paper, the evolution of the city interaction network is considered from the perspective of the edge. Therefore, when an edge is added to the network, its source node and the target node will be selected according to a certain mechanism. This selection mechanism is called preferential attachment of the edge. Here, six different preferential attachment models are considered in this paper:

**Random-Random (RR)**: for the arrived edge at time *t*, two nodes are randomly selected from the node set *V* as its source node and the target node, respectively:

(5) $$P_{RR}\left(z_{u,v}^{t}\right)=\frac{1}{N_{t}^{2}}.$$

**Random-Degree (RD)**: for the arriving edge at time *t*, a node is randomly selected from the node set *V* as its source node, and the selection of its target node is proportional to the degree of nodes in the network:

(6) $$P_{RD}\left(z_{u,v}^{t}\right)=\frac{{\left[k_{t}\left(v\right)\right]}^{\alpha }}{N\sum_{i\in V}{\left[k_{t}\left(i\right)\right]}^{\alpha }}.$$

**Degree-Random (DR)**: for the arrived edge at time *t*, a node is randomly selected from the node set *V* as its target node, and the selection of its source node is proportional to the degree of nodes in the network:

(7) $$P_{DR}\left(z_{u,v}^{t}\right)=\frac{{\left[k_{t}\left(u\right)\right]}^{\beta }}{N\sum_{i\in V}{\left[k_{t}\left(i\right)\right]}^{\beta }}.$$

**Geographical distance (G)**: for the arrived edge at time *t*, the selection of its source node and target node is proportional to the geographical distance between the two nodes:

(8) $$P_{G}\left(z_{u,v}^{t}\right)=\frac{{\left[d\left(u,v\right)\right]}^{\gamma }}{\sum_{i,j\in V}{\left[d\left(i,j\right)\right]}^{\gamma }}.$$

**Degree-Degree (DD)**: for the arrived edge at time *t*, the selection of its source node and target node is proportional to the degree of the nodes in the network. The degree index for the source node is α, and the degree index for the target node is β:

(9) $$P_{DD}\left(z_{u,v}^{t}\right)=\frac{{\left[k_{t}\left(v\right)\right]}^{\alpha }{\left[k_{t}\left(u\right)\right]}^{\beta }}{\sum_{i,j\in V}{\left[k_{t}\left(i\right)\right]}^{\alpha }{\left[k_{t}\left(j\right)\right]}^{\beta }}.$$

**Degree-Degree-Geographical distance (DDG)**: for the arrived edge at time *t*, the selection of its source node and target node is proportional to the degree of the nodes in the network and to the geographical distance between the source node and the target node. The degree index for the source node is α; the degree index for the target node is β; and the distance index is γ:

(10) $$P_{DDG}\left(z_{u,v}^{t}\right)=\frac{{\left[k_{t}\left(v\right)\right]}^{\alpha }{\left[k_{t}\left(u\right)\right]}^{\beta }{\left[d\left(u,v\right)\right]}^{\gamma }}{\sum_{i,j\in V}{\left[k_{t}\left(i\right)\right]}^{\alpha }{\left[k_{t}\left(j\right)\right]}^{\beta }{\left[d\left(i,j\right)\right]}^{\gamma }}.$$

## 4. Evaluation

In this section, a quantitative approach is applied to compare the accuracies of different preferential attachment models. The network is often considered to be the result of an evolutionary random process that drives its growth, including new nodes and new edges [35]. Given real data about network evolution, the extent to which the assumptions of a model are supported by the data using the maximum likelihood approach can be tested. The maximum likelihood approach is usually used to compare a series of models numerically and to select the best model (and parameters) to interpret the data. Estimating the likelihood of a preferential attachment model *M* involves considering each arriving edge $z^{t}$ and computing the likelihood $P_{M}\left(z_{u,v}^{t}\right)$ that the edge $z^{t}$ selects the actual source node *u* and the actual target node *v* according to the model *M*. Therefore, the likelihood of network $G_{T}$ generated by model *M* can be expressed as:

(11) $$P_{M}\left(G_{T}\right)=\prod_{t\in T}P_{M}\left(z_{u,v}^{t}\right).$$

To obtain better numerical accuracy, the log-likelihood is used in this paper:

(12) $$log\left(\prod_{t}P_{M}\left(z_{u,v}^{t}\right)\right)=\sum_{t}log\left(P_{M}\left(z_{u,v}^{t}\right)\right).$$

**Figure 5:**
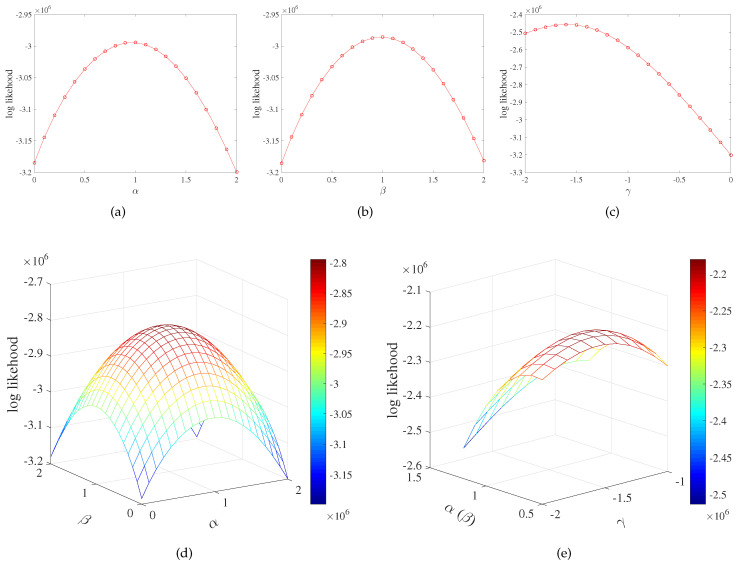
The relationship between log-likelihood of models and different parameters. (**a**) The relationship between the log-likelihood of the Random-Degree (RD) model and parameter α. (**b**) The relationship between the log-likelihood of the Degree-Random (DR) model and parameter β. (**c**) The relationship between the log-likelihood of the Geographical distance (G) model and parameter γ. (**d**) The relationship between the log-likelihood of the Degree-Degree (DD) model and parameters α and β. (**e**) The relationship between the log-likelihood of the Degree-Degree-Geographical distance (DDG) model and parameters α(β) and γ.

Since the city interaction network had self-connecting edges, which represents the interaction in the same city, we assumed that the distance of self-connecting edges was 20 kilometers (consider each city contour as a circle, and 20 kilometers is the approximate average of the radius of all cities). Figure 5 shows the relationship between the log-likelihood of models and different parameters. The RR model had no parameters, and its log-likelihood was a constant −3,185,899. In addition to the RR model, the log-likelihoods of the other five models were all convex functions of the model parameters, so the maximum likelihood of each model can be found to estimate the best parameters of the model. Table 3 lists the maximum log-likelihood of different preferential attachment models and the optimal parameters under the maximum log-likelihood. It can be seen from Figure 5 that, under the same parameter, the log-likelihood of the RD model and DR model was approximately equal. This reflects that the RD model and DR model had similar effects on the network evolution, and the selection of the source node and the target node was equal. Figure 5d also reflects this point. Figure 5c shows the relationship between the log-likelihood and parameter γ of G model, and its maximum log-likelihood was significantly higher than that of the RR model, RD model, DR model, and DD model, indicating that the distance played an important role in the evolution of the city interaction network. The DDG model considered both the node degree and the geography distance among nodes in the network evolution process. It can be seen that the maximum log-likelihood of DDG model was the highest, which was 22% higher than that of the DD model and 11% higher than that of the G model. In addition, in the DD model, when α=1.0, β=1.0, its log-likelihood was the maximum. In the G model, when γ=−1.6, its log-likelihood was the maximum. The DDG model, which considered the node degree and the geography distance, obtained the maximum likelihood when α=0.6, β=0.6, γ=−1.5. This indicates that the distance made the degree of the node less important. Then, we applied the DDG preferential attachment model with parameters α=0.6, β=0.6, γ=−1.5 to the evolution of the city interaction network.

## 5. Network Evolution

In order to verify the city interaction network model and the evolution process of the network, network evolution experiments were conducted. We considered the real network $G_{3T/4}$ from 2–4 July 2016 and evolved it from $t=\frac{3}{4}T$ until t=T. Specifically, the edge arrival model was used to determine the edges arriving at time $t\in [\frac{3}{4}T,T]$. For each arriving edge, the DDG preferential attachment model was used to select its source node and target node. Finally, the evolutionary network $G_{T}^{{}^{\prime }}$ with the same time length as the real network $G_{T}$ was obtained. $G_{T}$ and $G_{T}^{{}^{\prime }}$ were analyzed by the comparison of the statistical characteristics and community structure of the network.

**Figure 6:**
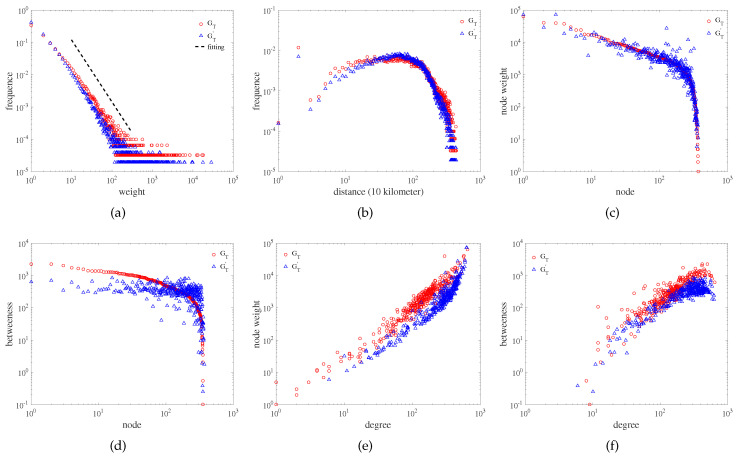
Statistical characteristics of real network $G_{T}$ and evolutionary network $G_{T}^{{}^{\prime }}$. (**a**) The weight distribution of edges. The weight distribution of real network $G_{T}$ is fitted as shown in the dotted black line. (**b**) The geography distance distribution of edges. The distance is in units of 10 kilometers. (**c**) The node weight distribution. The horizontal ordinate is the node number, and the numbering order is arranged in descending order of node weight. (**d**) The betweenness centrality distribution of nodes. The horizontal ordinate is the node number, and the numbering order is arranged in descending order of the betweenness centrality of nodes. (**e**) The relationship between node degree and node weight. (**f**) The relationship between node degree and node betweenness centrality. In the figure, the red circle marks represent the statistical characteristics of the real network $G_{T}$, and the blue triangle marks represent the statistical characteristics of the evolutionary network $G_{T}^{{}^{\prime }}$. All subgraphs are plotted on log-log coordinates.

Figure 6 shows the statistical characteristics of real network $G_{T}$ and evolutionary network $G_{T}^{{}^{\prime }}$. Figure 6a,b are considered from the edge properties. Figure 6a shows the weight distribution of the edges. It can be seen that the weight distributions of the real network and the evolutionary network followed the power-law distribution. The weight distribution of real network $G_{T}$ was fitted as shown in the dotted black line. The power exponents of the weight distributions of real network and evolutionary network were 1.92 and 1.99, respectively (the weight distributions of the real network and evolutionary network approximately overlapped, so the fit line of the weight distribution of the evolutionary network is not drawn). The weight of the edge represents the interaction among cities, and the power-law distribution of the weight distribution reflects that only a few cities had frequent interactions, while the interactions among most cities was very small. Figure 6b shows the geographical distance distribution of edges. The geographical distance distribution of edges is a property that connects the network with geographical space. Most of the interactive distances among cities were about 100 km. As the distance continued to increase, the probability of interaction became smaller. In addition, 20 km was also the high-frequency distance of city interaction (the distance was denoted as 20 km if the interaction occurred in the same city), indicating that the interaction in the same city occupied a large proportion.

Figure 6c–f are considered from the perspective of node properties. Figure 6c shows the node weight distribution; the horizontal ordinate is the node number, and the numbering order is arranged in descending order of node weight. The node weight of a node is the sum of all the weights of edges connected with the node, which reflects the interactions between the node and its neighbor nodes. Figure 6d shows the betweenness centrality distribution of nodes; the horizontal ordinate is the node number, and the numbering order is arranged in descending order of the betweenness centrality of nodes. The betweenness centrality is to measure the importance of a node to connect with other nodes. By comparing the real network $G_{T}$ with the evolutionary network $G_{T}^{{}^{\prime }}$, it can be found that the node weight and betweenness centrality of some nodes in the evolutionary network were obviously higher or lower than the real network, but the overall trend was consistent with the real network. The provincial capital is the economic, political, and cultural center of a province, which is also reflected in the city interaction network. In the real network shown in Figure 6c,d, provincial capitals have relatively high node weight and betweenness centrality, such as Beijing, Shanghai, Guangzhou, Suzhou, Tianjin, and Hangzhou, which can also be reflected in the evolutionary network. Figure 6e shows the relationship between node degree and node weight. Figure 6f shows the relationship between node degree and node betweenness centrality. The greater the degree of nodes, the greater the node weight and the betweenness centrality.

For the real network $G_{T}$ and evolutionary network $G_{T}^{{}^{\prime }}$, two community detection methods, Louvain [40] and Infomap [41], were used to extract the community structure of the network, and the Normalized Mutual Information (NMI) was used to evaluate the results of community detection. The evaluation results are shown in Table 4. $G_{T}-PAD$ represents the comparison between the community structure of real networks and the provincial administrative divisions in China; $G_{T}-G_{T}^{{}^{\prime }}$ represents the comparison between the community structure of the real network and that of the evolutionary network. It can be found that the community structure of the real network was consistent with the administrative division to a certain extent, and it also shows the influence of the distance factor on the interactions among cities. In addition, the community structure of evolutionary network and real network was also similar, which indicates that the preferential attachment model in this paper can describe the emergence of community to a certain extent. This is mainly because the distance factor was considered in the model, so that cities in the same province were easily connected and formed communities. In general, the evolutionary network can be well matched with the real network, which reflects that the model can not only capture the properties of the real city interaction network, but also reflect the geographical characteristics of the interactions among cities.

## 6. Discussion

### 6.1. Potential Biases

In this paper, the evolution of the city interaction network was modeled and analyzed by using the interactive data formed in the process of information dissemination. There is no doubt that the use of one dataset to explain the results is not complete enough. Since our model was data-driven, the edge arrival model and maximum likelihood method were data-dependent. For the edge arrival model, different spatial interactive data may have different situations. The selection of model parameters in this paper was based on the method of maximum likelihood. The optimal parameters of the model can be found using real data. Therefore, different datasets will lead to different optimal parameters of the model. The evolution model was evaluated by comparing the structure characteristics of the evolutionary network and the real network. From the results, the model can capture the properties of the real city interaction network, but this is only limited to the city interaction network formed in the process of information dissemination. In the process of information dissemination, the interaction of information enables people to express their emotions and opinions. It is helpful to understand people’s emotional tendency by considering the semantic characteristics of interactive information in the spatial interaction network.

Moreover, compared with cities in other countries, Chinese cities have some specificities. (1) China is a vast country, and the distance between cities is relatively large, making distance factors play an important role in the interactions of cities. (2) The distribution of Chinese cities shows a convergent pattern, which is different from Western countries. As a result, China has many large cities with large populations, such as Beijing, Shanghai, and Guangzhou. (3) The provincial administrative divisions in China are established around large cities, and the cities within the province are more likely to interact. The higher the level of political and economic development of the city, the more obvious the interaction. (4) China has a large population and a high Internet penetration rate, which makes information spread rapidly and widely. The results of this paper were obtained in this context. However, if the background were changed to some countries with a relatively small scale and the development levels of cities within the country were similar to each other, the influence of the distance factor on the interactions among cities may not be well reflected. Therefore, different countries have influence on the settings of the model.

### 6.2. Model Extension

The preferential attachment model in this paper belongs to a link prediction model based on the similarity of the network structure. Essentially speaking, a model for link prediction makes a guess about the factors resulting in the existence of links, which is actually what an evolving model wants to show. Up to now, the studies of link prediction overwhelmingly emphasized undirected networks. However, the study of link prediction in directed networks is inadequate [42].

The current common method for extending the technology applied to undirected networks to directed networks is to divide the degrees into outdegree and indegree, such as community detection [43,44,45,46]. According to this ideas, our model can be extended to directed networks. Take the DDG model as an example: the model can be extended to a directed network:

Directed-Degree-Degree-Geographical distance (DiDDG): for the arriving edge at time *t*, the selection of its source node is proportional to the out-degree of the nodes in the network; the selection of its target node is proportional to the in-degree of the nodes; meanwhile, the selection of its source node and target node is proportional to the geographical distance between the source node and the target node. The degree index for the source node is α; the degree index for the target node is β; and the distance index is γ:

(13) $$P_{DiDDG}\left(z_{u,v}^{t}\right)=\frac{{\left[k_{t}^{out}\left(v\right)\right]}^{\alpha }{\left[k_{t}^{in}\left(u\right)\right]}^{\beta }{\left[d\left(u,v\right)\right]}^{\gamma }}{\sum_{i,j\in V}{\left[k_{t}^{out}\left(i\right)\right]}^{\alpha }{\left[k_{t}^{in}\left(j\right)\right]}^{\beta }{\left[d\left(i,j\right)\right]}^{\gamma }}.$$

In the modified model, the degree is divided into the out-degree and in-degree for consideration, so that the probability of connecting an edge between node *u* and node *v* will vary depending on the direction of the edge.

## 7. Conclusions

This paper studied the evolution mechanism of the city interaction network formed in the process of information dissemination in social networks, where nodes represent cities and edges represent interactions among cities. We considered the evolution model of the city interaction network from the perspective of the edge. In the model, the nodes were fixed, and the evolution process of the edge consisted of two core processes: the edge arrival and the preferential attachment of the edge. The model of edge arrival determines the arrival time of each edge; the model of preferential attachment of the edge determines the source node and the target node of each arriving edge. Six preferential attachment models were considered, and the comparison was done by the maximum likelihood approach. We found that the degree of the node and the geographic distance of the edge were the key factors affecting the evolution of the city interaction network. The DDG preferential attachment model, which considered both the node degree and the geographical distance among nodes in the network evolution process, was the best of the six models. Finally, we conducted the evolution experiments using the most optimal model and compared it with the real city interaction network extracted from the information dissemination data of the WeChat web page. By comparing the weight, geographical distance, node weight, and betweenness centrality of the real network and the evolutionary network, it was found that the evolutionary network could be well matched to the real network, which reflects that the model can describe the actual characteristics of the interactions among cities. Our research is of great significance for providing location-based business services, planning and managing communication network facilities, and formulating regional economic development policies.

However, there are still some limitations in our work. On the one hand, the evolution process of the city interaction network is affected by a variety of factors, such as politics, economy, population, etc. A comprehensive comparative analysis of the effects of these factors plays a significant role in the evolution model. These factors should be considered in the evolution model in future work. On the other hand, our work was verified by the real dissemination data of the WeChat web page; whether the model is applicable to the evolution of other spatial interaction networks still needs to be further verified.

## Figures and Tables

**Table 1:** City distribution of 34 provincial divisions in China. China has 34 provincial divisions, including 23 provinces, 4 municipalities, 5 autonomous regions, and 2 special administrative regions.

Province	Number of Cities	Province	Number of Cities	Province	Number of Cities
Beijing	1	Tianjin	1	Hebei	11
Inner Mongolia	12	Liaoning	14	Jilin	9
Shanghai	1	Jiangsu	13	Zhejiang	21
Fujian	16	Jiangxi	9	Shandong	11
Hubei	18	Hunan	17	Guangdong	14
Hainan	18	Chongqing	1	Sichuan	21
Yunnan	16	Xizang	7	Shannxi	10
Qinghai	8	Ningxia	5	Xinjiang	15
Shanxi	11	Heilongjiang	13	Anhui	11
Henan	17	Guangxi	14	Guizhou	9
Gansu	14	Hong Kong	1	Macao	1
Taiwan	12				

**Table 2:** Basic properties of the city interaction network $G_{T}$. *N* represents the number of nodes, $M_{T}$ the number of edges, $M_{T}^{se}$ the number of self-connected edges, $k_{T}^{avg}$ the average degree of nodes, $\rho_{T}$ the density, and $L_{T}$ the average length of the shortest path.

*T*	*N*	$M_{T}$	$M_{T}^{\mathrm{se}}$	$k_{T}^{\mathrm{avg}}$	$\rho_{T}$	$L_{T}$
2–8 July 2016	372	30,438	353	163.65	0.22	1.73

**Table 3:** The maximum log-likelihood of different preferential attachment models and the optimal parameters under the maximum log-likelihood.

Model	Parameter	The Maximal Log-Likelihood
RR	-	−3,185,899
RD	α=1.0	−2,994,407
DR	β=1.0	−2,985,583
G	γ=−1.6	−2,456,443
DD	α=1.0	−2,794,647
	β=1.0	
DDG	α=0.6	−2,180,441
	β=0.6	
	γ=−1.5	

**Table 4:** Evaluation results of community detection in undirected networks. $G_{T}$ represents the real network, PAD represents Provincial Administrative Divisions in China, and $G_{T}^{{}^{\prime }}$ represents the evolutionary network.

Comparison	Louvain	Infomap
$G_{T}-PAD$	0.738	0.831
$G_{T}-G_{T}^{{}^{\prime }}$	0.715	0.850
